# A phantom study of the immobilization and the indications for using virtual isocenter in stereoscopic X‐ray image guidance system referring to position localizer in frameless radiosurgery

**DOI:** 10.1120/jacmp.v14i4.4133

**Published:** 2013-07-08

**Authors:** Hsiao‐Han Chang, Hsiao‐Fei Lee, Chien‐Cheng Sung, Tsung‐I Liao, Yu‐Jie Huang

**Affiliations:** ^1^ Department of Radiation Oncology Kaohsiung Chang Gung Memorial Hospital and Chang Gung University College of Medicine Niao‐Sung Kaohsiung 833 Taiwan; ^2^ Bio‐Image and Electronics Laboratory Department of Biomedical Engineering, National Cheng Kung University Tainan 701 Taiwan

**Keywords:** frameless, radiosurgery, immobilization, image guidance, virtual isocenter, position localizer

## Abstract

A frameless radiosurgery system is using a set of thermoplastic mask for fixation and stereoscopic X‐ray imaging for alignment. The accuracy depends on mask fixation and imaging. Under certain circumstances, the guidance images may contain insufficient bony structures, resulting in lesser accuracy. A virtual isocenter function is designed for such scenarios. In this study, we investigated the immobilization and the indications for using virtual isocenter. Twenty‐four arbitrary imaginary treatment targets (ITTs) in phantom were evaluated. The external Localizer with positioner films was used as reference. The alignments by using actual and virtual isocenter in image guidance were compared. The deviation of the alignment after mask removing and then resetting was also checked. The results illustrated that the mean deviation between the alignment by image guidance using actual isocenter (${\text{Iso}}_{\text{img}}$) and the localizer(${\text{Iso}}_{\text{loc}}$) was 2.26mm±1.16mm (standard deviation, SD), 1.66mm±0.83mm for using virtual isocenter. The deviation of the alignment by the image guidance using actual isocenter to the localizer before and after mask resetting was 7.02mm±5.8mm. The deviations before and after mask resetting were insignificant for the target center from skull edge larger than 80 mm on craniocaudal direction. The deviations between the alignment using actual and virtual isocenter in image guidance were not significant if the minimum distance from target center to skull edge was larger or equal to 30 mm. Due to an unacceptable deviation after mask resetting, the image guidance is necessary to improve the accuracy of frameless immobilization. A treatment isocenter less than 30 mm from the skull bone should be an indication for using virtual isocenter to align in image guidance. The virtual isocenter should be set as caudally as possible, and the sella of skull should be the ideal point.

PACS numbers: 87.55.kh, 87.55.ne, 87.55.tm

## I. INTRODUCTION

Radiosurgery is a delicate treatment to deliver highly precise radiation to a target. For precision, the standard immobilization in radiosurgery is frame‐based with metal pins on the skull.^(1)^ Although the frame‐based radiosurgery provides high accuracy, the disadvantages of head rings are pain and general discomfort. It is also mainly limited to single‐fraction treatments of the invasive nature, and a trend toward noninvasive frameless stereotactic systems has developed. The patient is immobilized by a set of thermoplastic mask, and improvements in image guidance have great potential to provide a precision treatment.^2^, ^3^, ^4^

**Figure 1:**
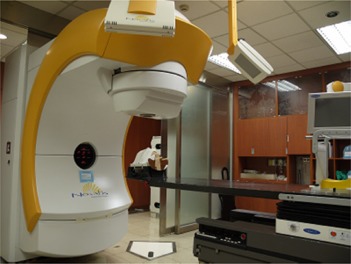
The Novalis with ExacTrac system. The phantom is set on Novalis ExtracTrac with frameless supporting system.

**Figure 2:**
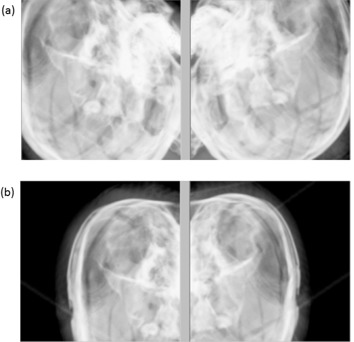
The stereotactic X‐ray images (a) for image guidance; (b) some images may not have sufficiency bony structure for image fusion. (There are also some structures from couch in the blank area.)

In our clinic, Novalis with ExacTrac (BrainLAB, Heimstetten, Germany, and Varian Associates, Palo Alto, CA) systems are used for frameless radiosurgery (Fig. 1). The system has been proven to be safe and reliable with an acceptable accuracy.^(5)^ However, there are uncertainties regarding the system in practice. First, thermoplastic mask is not as rigid as the frame‐based immobilization. Second, the accuracy of image guidance depends on fusing the stereoscopic X‐ray images to digital radiography reconstruction (DRR) images by planning system. Due to the geometric set up of the stereoscopic X‐rays and the fact that the isocenter of the Novalis gantry is fixed, the patient is set up to fit the treatment isocenter. Under certain circumstances, DRR images are centered at the point that does not include sufficient bony structures, such as meningeal lesions located beside the skull bone (Fig. 2). The accuracy will be less due to lack of image information. Besides, the blank area of the X‐ray image may contain the object of couch that did not exist in CT simulation images for DRR in fusion that may affect the fusion result. The virtual isocenter with definite shifts from the actual isocenter for verification purposes could be a solution. Unfortunately, there are no indications available in the literature about using virtual isocenter.

The BrainLab Novalis provides a frameless system that has both a Head And Neck Localizer for laser‐based stereotactic localization and a frameless radiosurgery‐positioning array in image guidance for alignment. In frame‐based treatment, laser alignment has a satisfactory precision.^(6)^ In image guidance, the system uses stereoscopic kV X‐rays with spatial uncertainty of less than 2 mm.^(7)^ The Head And Neck Localizer, which is made of acrylic glass with six embedded localizer rods, defines a precise, stereotactic, three‐dimensional coordinate within the patient's cranial volume on CT images. The localizer establishes coordinates of the treatment volume with accuracy and precision. The BrainLab treatment planning system establishes the space matrix by the Localizer and presents the location of treatment isocenter on target positioner films. Therefore, the planned isocenter in CT coordinates is given on the form cross‐line on target positioner films. The target positioner films are used to indicate the isocenter that is identified by treatment planning system. The image guidance system should align the treatment volume to the isocenter. Therefore, the error of image guidance should be established according the deviation of laser projection after regulating the laser with Winston‐Lutz tests.^(8)^

In this study, we evaluate the representation of frameless immobilization and the indications for using virtual isocenter by taking the Head And Neck Localizer and the Target Positioner as reference in phantom.

## II. MATERIALS AND METHODS

### A. Treatment system and calibration

Winston‐Lutz tests^(8)^ were performed before the procedures with an acceptable limit of <1mm to confirm that the radiation isocenter of was confined to the mechanical isocenter of gantry and laser crosshairs. Infrared red (IR) camera calibration, isocenter calibration, and X‐ray calibration for image guidance were also performed before the examinations.

### B. Phantom and support system

Cranial portion of RANDO phantom (The Phantom Laboratory, Salem, NY) was used for simulation. The phantom was immobilized by BrainLAB Frameless Radiosurgery Mask Set modeling according to the procedures on BrainLAB manual.

### C. Computer tomography scanning for treatment planning

The phantom was set up on General Electric (GE) LightSpeed RT unit (GE Medical Systems, Milwaukee, WI) with BrainLAB frameless radiosurgery support system and Head and Neck Localizer. The phantom was scanned helically with thickness of 1.25 mm. After scanning, the frameless mask was not removed for excluding the factor of mask resetting in following examinations. The images were export to treatment planning system.

### D. Treatment planning system and imaginary targets for examination

The scanned images were imported into BrainLAB ImageRT version 3.0. After localization with BrainLAB Head and Neck Localizer, arbitrary imaginary treatment targets (ITTs) were delineated in phantom images. The distance of ITT isocenter from outer skull bone according to x‐axis (right–left), y‐axis (ventral–dorsal), and z‐axis (caudal–cranial) were documented. The target positioner films were printed by BrainLAB BrainSCAN version 5.0, and the treatment target centered DRRs were exported to ExacTrac by Brain iPlan RT Dose version 3.0, according to individual ITTs for image guidance alignment verification.

### E. Alignment using actual isocenter

**Figure 3:**
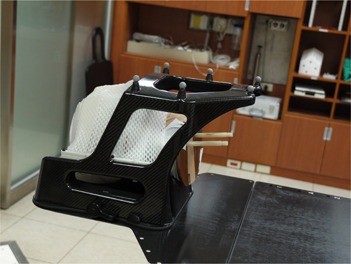
Attaching Radiosurgery Positioning Array to the base plate for optic guide prepositioning.

The phantom was set up on the treatment table (Fig. 1). The BrainLAB Frameless Radiosurgery Positioning Array was attached to the base plate for prepositioning (Fig. 3). X‐ray correction was then performed. Two X‐ray images were taken, bony image fusion was conducted, and the fusion shift was determined. The couch was moved to the treatment position with a Novalis Body/ExacTrac Robotic Tilt Module.

**Figure 4:**
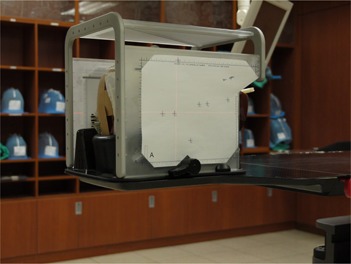
After image guidance, the laser crosshair is projected on Head and Neck Localizer and Target Positioner for recording the setup deviation from center of the target positioner films.

At this time, the phantom was placed in the treatment position. The Head and Neck Localizer and Target Positioner with target positioner films were then attached to the base plate instead of the Frameless Radiosurgery Positioning Array. The laser crosshair projections on each plane of the Head and Neck Localizer and Target Positioner were documented on the target positioner films (Fig. 4).

### F. Alignment using virtual isocenter

**Figure 5:**
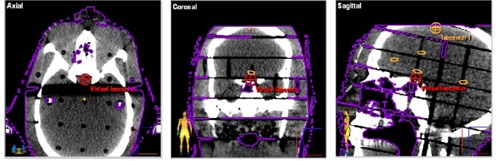
The virtual isocenter was defined in the center of skull (i.e., sella) for alignment.

A certain virtual isocenter was defined in the geometric center of the phantom skull (i.e., sella (Fig 5.)). Because the ITTs were defined in the same CT image volume and the same treatment planning, the virtual was constant for each ITT. The offsets of the actual isocenter and the virtual center were defined and taken into consideration by the system. The X‐ray correction procedures were aligned to the virtual isocenter, but the correction shift to the actual isocenter was calculated and corrected. After the phantom was moved to the treatment position, the documented procedures on the target positioner films were identical to those used in the alignment using actual isocenter.

### G. Assessment of the spatial error of alignment in image guidance regarding to the Head and neck Localizer

The Head and Neck Localizer and Target Positioner were simplified into a cuboid. The ITT isocenter was projected onto the external planes of the Localizer with target positioner films from the planning system. In other words, the Localizer cuboid established a coordinate space from the CT scanning volume for treatment with the origin at the isocenter and the cross‐lines on the target positioner films indicated the treatment isocenter according to initial CT images for treatment planning, ${\text{Iso}}_{\text{loc}}$, by the output of treatment planning. The crosshair lasers in the treatment room indicated the origin of the linear accelerator gantry isocenter, which should be identical to the treatment isocenter ideally after Winston‐Lutz test. After alignment with image guidance, the laser crosshairs were projected onto the A, B, and C planes of target positioner films on the Head and Neck Localizer. The laser crosshairs may have offset from the printed cross‐line on the target positioner films. On plane A, the offset according to the y‐ and z‐axes was measured using a vernier for $y_{A}$ and $z_{A}$. The parameters of $x_{B},z_{B},y_{C}$, and $z_{C}$ were caught on planes B and C. However, the side lengths of the Head and Neck Localizer and Target Positioner were identical, and the parameter $x_{A}$ could be measured according to the distance from the projected point on the B plane to the wedge of the A plane, as well as $y_{B}$ and $x_{C}$. To summarize, the relative coordinates for the laser crosshair projection points to the printed cross‐line on the target positioner films on planes A, B, and $C—P_{A}(x_{A},y_{A},z_{A}),P_{B}(x_{B},y_{B},z_{B})$, and $P_{C}(x_{C},y_{C},z_{C}$), respectively — could be identified.

**Figure 6:**
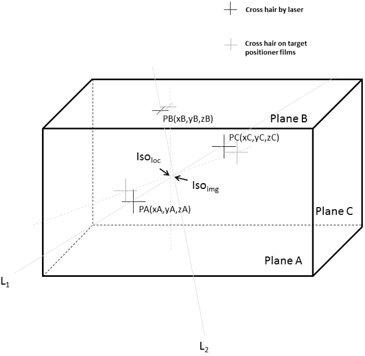
The relation of the isocenter by image guidance and laser crosshair. After image guidance, the laser crosshair is projected onto the Head and Neck Localizer and Target Positioner to record the setup deviation from the isocenter of the target positioner films. The coordination is established by the Head and Neck Localizer and Target Positioner ${\text{Iso}}_{\text{loc}}$. The laser crosshair indicates the ${\text{Iso}}_{\text{img}}$. The guides are used to solve the position of ${\text{Iso}}_{\text{img}}$.

As shown in Fig. 6, the isocenter by image guidance, ${\text{Iso}}_{\text{img}}$, was on the line that was established between $P_{A}$ and $P_{C},L_{1}$. The equation of the line can be expressed with relative coordinates as follows:

(1) $$\begin{array}{l} x=x_{A}+(x_{C}-x_{A})t\\ y=y_{A}+(y_{C}-y_{A})t\\ z=z_{A}+(z_{C}-z_{A})t \end{array}$$

where each of *(x, y, z)* is an arbitrary point of the line, and *t* is a real number.

The line $L_{2}$, between $P_{B}$ and ${\text{Iso}}_{\text{img}}$, was a normal line to $L_{1}$, which can be illustrated as follows:

(2) $$(x_{C}-x_{A})x+(y_{C}-y_{A})y+(z_{C}-z_{A})z=d$$

where (x, y, z) is an arbitrary point of the line, and *d* is a constant.

The line $L_{2}$ passes through point $P_{B}$ on plane B. Therefore, the constant, d, could be solved by substitution of the $P_{B}(x_{B},y_{B},z_{B})$ into the equation for $L_{2}$.

Finally, by substituting Eq. (1) for Eq. (2), t could be obtained. The point of intersection of $L_{1}$ and $L_{2}$ is the isocenter by image guidance ${\text{Iso}}_{\text{img}}$. The relative coordinate is the deviation of the isocenter by image guidance and the isocenter established by treatment planning system which is projected on the Head and Neck Localizer, ${\text{Iso}}_{\text{loc}}$ (i.e., the error of image guidance).

### H. Experiment design

Twenty‐four ITTs were arbitrarily denoted and checked. The phantom was delivered to the Novalis treatment room without removing the mask for excluding the setup error after CT scanning. Every isocenter was aligned with image guidance using the actual isocenter and the virtual isocenter. Then, the mask of the phantom was removed and set up again, alignment using actual isocenter to evaluate the frameless system resetup error, according to every ITT.

### I. Statistics

The means and standard deviations of the distances of ${\text{Iso}}_{\text{img}}$ and ${\text{Iso}}_{\text{loc}}$ were calculated. A paired *t*‐test was used to check the significance between the alignment using actual and virtual isocenter, by the isocenter without and with mask resetting, and by the isocenter of each ITT group. We assessed a p‐value <0.05 as statistically significant.

## III. RESULTS

### A. The deviation of ${\text{Iso}}_{\text{img}}$ and ${\text{Iso}}_{\text{loc}}$ by the alignment

**Figure 7:**
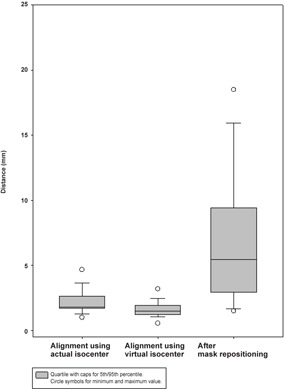
The quartile box plot of the deviations of ${\text{Iso}}_{\text{loc}}$ and ${\text{Iso}}_{\text{img}}$ by the alignment using actual ioscenter, virtual isocenter, and after mask resetting.

The mean distance ± standard deviation (SD) of ${\text{Iso}}_{\text{img}}$ and ${\text{Iso}}_{\text{loc}}$ was 2.26mm±1.16mm by alignment using actual isocenter, 1.66mm±0.83mm by alignment using virtual isocenter, and 7.02mm±5.8mm after mask resetting aligned using actual isocenter. The quartile box plots are shown in Fig. 7. There was a statistical significance between the alignment using actual and virtual isocenter, with a p‐value of 0.001. The deviations between with and without mask resetting by alignment using actual isocenter were also significant, with a p‐value of <0.001. Considering the deviation of ${\text{Iso}}_{\text{img}}$ and ${\text{Iso}}_{\text{loc}}$ according to different axis directions compared to the alignment using actual isocenter, statistical significance existed only on the y‐axis (ventral–dorsal) after mask resetting, with a p‐value of <0.001. The results of the deviation of ${\text{Iso}}_{\text{img}}$ and ${\text{Iso}}_{\text{loc}}$ by alignment using virtual isocenter and after mask resetting compared to alignment using actual isocenter are shown in Table 1.

**Table 1:** A summary of the deviation of ${\text{Iso}}_{\text{img}}$ and ${\text{Iso}}_{\text{loc}}$

*Check Item*	Mean±standard Deviation(SD)	*Paired* t‐*test p‐value Compared to Alignment Using Actual Isocenter*
Alignment by Actual Isocenter		
x‐axis	−0.59±1.00mm	
y‐axis	0.50±1.06mm	
z‐axis	−0.83±1.79mm	
Distance	2.26±1.16mm	
Alignment by Virtual Isocenter		
x‐axis	−0.61±0.66mm	0.880
y‐axis	0.60±0.51mm	0.618
z‐axis	−0.61±1.31mm	0.458
Distance	1.66±0.83mm	0.001^a^
Alignment After Mask Resetting Using Actual Isocenter		
x‐axis	−1.32±4.08mm	0.368
y‐axis	−2.29±1.23mm	<0.001 ^a^
z‐axis	−0.16±7.73mm	0.668
Distance	7.03±5.81mm	<0.001 ^a^

a p<0.05

### B. The deviation of ${\text{Iso}}_{\text{img}}$ and ${\text{Iso}}_{\text{loc}}$ with/without mask re‐setting

Table 2 illustrates the deviation of ${\text{Iso}}_{\text{img}}$ and ${\text{Iso}}_{\text{loc}}$ without and after mask resetting aligned using actual isocenter according to axes. The results indicate that the deviation after masking resetting was statistically significant regardless of the distance from the ITT isocenter to the skull edge on the x‐ (right–left) and y‐axes (ventral–dorsal) and the minimal distance. Only when the distance of the ITT isocenter and the skull edge was greater than 80 mm on the z‐axis (cranial–caudal) after mask resetting became statistically insignificant, but the deviations still showed a trend, with a p‐value less than 0.1.

**Table 2:** Paired *t*‐test of the deviation of ${\text{Iso}}_{\text{img}}$ and ${\text{Iso}}_{\text{loc}}$ between without and with mask resetting aligned using actual isocenter

*ITT Isocenter to the Skull Edge*	*Number* (N=24)	Mean±standard Deviation(SD)(mm) of ${\text{Iso}}_{\text{img}}$ *and* ${\text{Iso}}_{\text{loc}}$ *(without mask resetting)*	Mean±standard Deviation(SD)(mm) *of* ${\text{Iso}}_{\text{img}}$ *and* ${\text{Iso}}_{\text{loc}}$ *(after mask resetting)*	*p‐value of Paired* t*‐test*
On x‐axis				
≥20mm	18	2.30±1.22	6.34±4.81	0.002^a^
≥3030	13	2.33±1.36	7.09±4.93	0.004 ^a^
≥4040	12	2.00±0.69	7.13±5.15	0.004 ^a^
≥5050	8	1.85±0.33	4.26±1.76	0.003 ^a^
On y‐axis				
≥3030	18	2.20±1.22	5.66±4.20	0.002 ^a^
≥4040	15	2.12±1.20	4.00±1.79	<0.001 ^a^
≥5050	13	2.18±1.28	4.02±1.93	0.002 ^a^
≥6060	8	1.86±0.33	4.32±1.81	0.003 ^a^
On z‐axis				
≥2020	21	2.00±0.72	7.37±60.9	<0.001 ^a^
≥3030	20	2.05±0.70	7.13±6.14	0.001 ^a^
≥4040	19	2.06±0.72	7.15±6.31	0.002 ^a^
≥5050	17	1.95±0.64	7.12±6.49	0.003 ^a^
≥6060	14	2.08±0.61	7.04±6.85	0.012 ^a^
≥7070	12	2.01±0.56	6.60±6.85	0.031 ^a^
≥8080	8	2.03±0.62	5.02±4.81	0.086
≥9090	7	1.85±0.39	3.46±2.08	0.055
Minimum Distance				
≥2020	17	2.07±0.73	6.32±4.96	0.001 ^a^
≥3030	11	1.89±0.60	6.33±4.55	0.007 ^a^
≥4040	9	1.84±0.31	4.42±1.72	0.001 ^a^
≥5050	8	1.85±0.33	4.26±1.76	0.003 ^a^

a p<0.05

### C. The ITT characteristics corresponding to alignment

Table 3 shows the characteristics from the isocenter to the skull edge on different axis and the minimum distance that correspond to the deviations between the alignment using actual and virtual isocenter. The results show that the deviations of ${\text{Iso}}_{\text{img}}$ and ${\text{Iso}}_{\text{loc}}$ between the alignment using actual and virtual isocenter became nonsignificant if the distance from the treatment isocenter to the skull edge was greater than or equal to 40 mm, 60 mm, and 80 mm on the x‐ (right–left), y‐ (ventral–dorsal), and z‐axis (cranial–caudal), respectively. Regardless of the axis direction, if the minimum distance of the treatment isocenter and skull edge was greater than or equal to 30 mm, the deviations of ${\text{Iso}}_{\text{img}}$ and ${\text{Iso}}_{\text{loc}}$ between the alignment using actual isocenter and virtual isocenter were not statistically significant.

**Figure 8:**
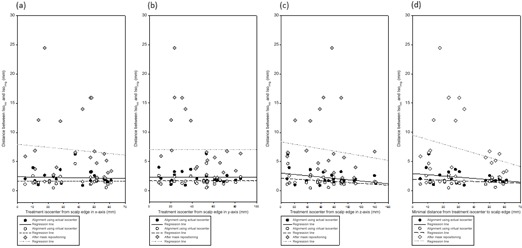
The scatter plots according to axes and minimum distance: (a) the distance of ${\text{Iso}}_{\text{loc}}$ and ${\text{Iso}}_{\text{img}}$ between the alignment using actual isocenter, virtual isocenter and after mask resetting vs. the treatment isocenter from the skull edge on the x‐axis (right–left) with regression lines; (b) the distance of ${\text{Iso}}_{\text{loc}}$ and ${\text{Iso}}_{\text{img}}$ between alignment using actual isocenter, virtual isocenter and after mask resetting vs. the treatment isocenter from the skull edge on the y‐axis (ventral–dorsal) with regression lines; (c) the distance of ${\text{Iso}}_{\text{loc}}$ and ${\text{Iso}}_{\text{img}}$ between alignment using actual isocenter, virtual isocenter and after mask resetting vs. the treatment isocenter from the skull edge on the z‐axis (cranial–caudal) with regression lines; (d) the distance of ${\text{Iso}}_{\text{loc}}$ and ${\text{Iso}}_{\text{img}}$ between alignment using actual isocenter, virtual isocenter and after mask resetting vs. the minimal distance of the treatment isocenter from the skull edge with regression lines.

Figure 8 show the scatter plots with the regression lines of the distance of ${\text{Iso}}_{\text{loc}}$ and ${\text{Iso}}_{\text{img}}$ and the distance from the treatment isocenter to the skull edge, according to the axis, and the minimal distance, grouped with alignment using actual isocenter, virtual isocenter, and after mask resetting. All of these plots demonstrate that the distances of ${\text{Iso}}_{\text{loc}}$ and ${\text{Iso}}_{\text{img}}$ were most unsatisfactory after mask resetting. The regression lines of the alignment using actual isocenter and virtual center converged as the distance from the treatment isocenter to the skull edge increased.

**Table 3:** Paired *t*‐test of the deviation of ${\text{Iso}}_{\text{img}}$ and ${\text{Iso}}_{\text{loc}}$ between the alignment using actual isocenter and virtual isocenter according to ITT characteristics

*ITT Isocenter to Skull Edge*	*Number* (N=24)	Mean±standard Deviation(SD)(mm) *of* ${\text{Iso}}_{\text{img}}$ *and* ${\text{Iso}}_{\text{loc}}$ *by the Alignment Using Actual Isocenter*	Mean±standard Deviation(SD)(mm) *of* ${\text{Iso}}_{\text{img}}$ *and* ${\text{Iso}}_{\text{loc}}$ *by the Alignment Using Virtual Isocenter*	*p‐value of Paired* t‐*test*
On x‐axis				
≥1010	n=23	2.27±1.19	1.68±0.84	0.001^a^
≥2020	n=18	2.3±1.22	1.68±0.92	0.004 ^a^
≥3030	n=13	2.33±1.36	1.80±1.00	0.033 ^a^
≥4040	n=12	2.00±0.69	1.56±0.53	0.069
≥5050	n=8	1.85±0.33	1.56±0.55	0.179
On y‐axis				
≥3030	n=18	2.20±1.22	1.63±0.91	0.008 ^a^
≥4040	n=15	2.12±1.20	1.61±0.97	0.014 ^a^
≥5050	n=13	2.18±1.28	1.65±1.04	0.022 ^a^
≥6060	n=8	1.86±0.33	1.62±0.44	0.206
On z‐axis				
≥2020	n=21	2.00±0.72	1.50±0.51	0.006 ^a^
≥3030	n=20	2.05±0.70	1.51±0.52	0.004 ^a^
≥4040	n=19	2.06±0.72	1.51±0.54	0.005 ^a^
≥5050	n=17	1.95±0.64	1.52±0.45	0.019 ^a^
≥6060	n=14	2.08±0.61	1.49±0.49	0.005 ^a^
≥7070	n=12	2.01±0.56	1.43±0.46	0.014 ^a^
≥8080	n=8	2.03±0.62	1.44±0.30	0.059
≥9090	n=7	1.85±0.39	1.47±0.31	0.081
Minimum Distance				
≥2020	n=17	2.07±0.73	1.51±0.57	0.008 ^a^
≥3030	n=11	1.89±0.60	1.49±0.49	0.119
≥4040	n=9	1.84±0.31	1.51±0.54	0.099
≥5050	n=8	1.85±0.33	1.56±0.55	0.179

a p<0.05

## IV. DISCUSSION

Radiosurgery refers to the precise delivery of radiation to a focal target.^(9)^ To achieve maximal accuracy, the patient‐supporting system should be very reliable, as should the neuroimaging. Conventionally, radiosurgery has used an invasive head frame with good accuracy.^1^, ^8^ However, invasive head frames are uncomfortable and not suitable for fractionated treatment. A noninvasive frameless system has been developed. The BrainLab Novalis with ExacTrac radiosurgery system provides a frameless radiosurgery system. It is using image‐guided positioning techniques. Gevaert et al.^(10)^ have developed a study to check the setup accuracy of the Novalis system. They applied computer program to rotate the reference CT data, compensate the established error with couch, and verify the positional accuracy with portal films. They got a subdegree of accuracy as conclusion. However, they did not describe how to represent the position of phantom from CT to treatment couch that takes place in real treatment scenario. Second, they emphasized the detection of rotation, but merely transposition. Finally, the error is established by portal films without evaluating the shooting angles’ correction. These could create systematic errors with a good precision in testing but a misleading accuracy.

The Novalis system provides both an external localizer for laser‐based alignment and a radiosurgery‐positioning array for image guidance alignment. In this study, we used the Head and Neck Localizer as the reference. Since the volume matrix is established by Localizer for treatment planning, the Localizer is the idea reference to check the accuracy. The lasers in treatment room are used to indicate the gantry isocenter and it is also required to be quality assured by Radiation Therapy Task Group 40 Report from the American Association of Physicists in Medicine (AAPM). Therefore, the Localizer with laser indication should be the absolute reference for checking. Furthermore, the masks on the phantom were not removed after CT imaging for examination of alignment using actual and virtual isocenter, which could be suggested to be a rigid body and exclude the error of setting up the mask. The space that was established by the Localizer was supposed to be the reference coordinates to check the accuracy of the frameless system.

Although the frameless supporting system uses a set of mask for tight fixation, the immobilization capability and overall accuracy have been considered to be less than an invasive frame‐based system.^11^, ^12^ The fixation (i.e., the mask) was removed after CT scanning and reset up before treatment in practical treatment process. It is difficult to represent the position exactly with the position in CT scanning. Although we used phantom that is more rigid than human in this study and the mask was not remove after CT scanning, the mask and phantom are still not an absolute rigid body. It is not possible to represent the position totally even in phantom. In our results, the mean distance of ${\text{Iso}}_{\text{img}}$ and ${\text{Iso}}_{\text{loc}}$ by the alignment using actual isocenter was 2.26mm±1.16mm without mask resetting, but 7.02mm±5.8mm after mask resetup. The deviations after mask resetting were significant, except the distance from treatment center to skull edge larger or equal to 80 mm on the z‐axis (cranial–caudal direction) (Table 2). This result may suggest that the more cephalic the lesion is, the worse the immobilization will be. This finding indicates that the immobilization may only be acceptable in the lower part of the cranium.

The development of image guidance has provided assistance for frameless radiosurgery.^13^, ^14^ The Novalis system utilizes stereoscopic kV X‐ray images through the machine isocenter.^(13)^ The system creates image fusion of the kV X‐ray images with DRR generated by the planning system to establish a predicted position shift for aligning the patient such that the target is coincident with the planning isocenter. An infrared tracking system is used to provide the initial patient position and to verify relative shifts. The system can be safely and reliably used as a target localization device with accuracy to within 1 mm by end‐to‐end phantom tests.^(5)^ However, the DRRs were generated from 1.25 mm CT slices in this study, and the mean deviations of ${\text{Iso}}_{\text{img}}$ and ${\text{Iso}}_{\text{loc}}$ aligned using actual isocenter, at 2.26mm±1.16mm, were acceptable.

However, clinical applications are more complex. The X‐ray tubes of Novalis system are embedded on floor and the detective panels are 25 cm by 25 cm mounted on the ceiling with about 2 m high. The stereoscopic kV X‐rays cross the isocenter of the gantry. The gantry isocenter is located about 1 m high from the floor. Therefore, the acquired image may be only about 12.5 cm by 12.5 cm of the skull. Besides, the isocenter is fixed and the patient is set up to isocenter. The treatment of lesions may be centered on DRRs without sufficient visible bony structures. The image guidance depends on the fusion of stereoscopic X‐ray and corresponding DRR images. The fusion result might not be reliable without sufficient information. There is an alternative method for verification. The virtual isocenter is an isocenter with recognized shifts from the planning isocenter to help the improvement of the image information in kV X‐ray fields, which would provide greater accuracy. The indications for defining a virtual isocenter in the BrainLAB user guide of the Novalis Body/ExacTrac are the following: cases that do not have sufficient visible bony structures, and cases only displaying a periodic structure which can be confused with similar structures. In cranial treatment scenarios, there are no periodic structures anatomically. The indications for using virtual isocenter in cranial treatment will be cases that centered without sufficient visible bony structures in image guidance X‐ray images.

It is difficult to define insufficient visible bony structures, and no reference is available. Because the skull is similar to a sphere, the visible parts on image depend on the X‐ray section to the skull edge. Therefore, it should be rational to use the distance between the treatment isocenter to the skull edge for the characteristics for the indication to align using virtual isocenter. In our results, the deviations of ${\text{Iso}}_{\text{img}}$ and ${\text{Iso}}_{\text{loc}}$ between the alignment using actual and virtual isocenter were significant for a short distance of the ITT isocenter and the skull edge. Table 3 indicates that the deviations of ${\text{Iso}}_{\text{img}}$ and ${\text{Iso}}_{\text{loc}}$ between the alignment using actual and virtual isocenter lost their power and significance when the distance from the ITT isocenter to the skull edge was larger or equal to 40 mm, 60 mm and 70 mm on the x‐ (right–left direction), y‐ (ventral–dorsal direction), and z‐axis (cranial–caudal direction), respectively. With regard to the minimum distance of the ITT isocenter to the skull edge, the critical distance is 30 mm.

Although the mean deviation was generally less in the alignment using virtual isocenter which might suggest a better accuracy, there is a warning about the alignment using virtual isocenter. The virtual isocenter is used only for verification purpose. Thus, using a virtual isocenter with shifts from the actual isocenter for alignment is suggested to be less accurate. Moreover, the procedures of the alignment using virtual isocenter are more complex, which require a longer setup time and might easily result in inaccurate procedures. It is suggested that using virtual isocenter for alignment should not be a routine procedure and should be used only in certain conditions. According to our results, a distance of the treatment isocenter to the skull edge less than 30 mm should be a criterion for introducing virtual isocenter for alignment, which could ensure that the accuracy error remains within the acceptable range.

On the other hand, the decision of the point for establishing the virtual isocenter is another issue. In the concept of image guidance discussed above, the more bony image information that is available, the more satisfactory the fusion obtained will be. The virtual isocenter should be located as caudally as possible to increase the bony portion of skull on image guidance X‐ray film. However, the cranial structure is nearly spherical, and the length of the Localizer is limited. According to the results of this study, immobilization is better, even after mask resetting, when the distance from the isocenter to the skull edge is greater than 80 mm on the z‐axis (i.e., cranial–caudal direction) (Table 2). Table 3 also demonstrates that the deviations of ${\text{Iso}}_{\text{img}}$ and ${\text{Iso}}_{\text{loc}}$ between the alignment using the actual and virtual isocenter were not significant once the distance from the skull edge to the isocenter was greater than 80 mm on the z‐axis (cranial–caudal direction). This result suggests that sella of the skull should be an ideal point for establishing virtual isocenter, and it should provide sufficient bony details for accurate image guidance.

Although frameless radiosurgery procedures are less invasive and more comfortable for the patient, there are more uncertainties in these procedures than in frame‐based radiosurgery. Frameless radiosurgery should be conducted attentively because it depends greatly on manipulative procedures. This study used a phantom to verify the alignment. The phantom is more rigid and motionless than human. The results of this study suggest the uncertainties of immobilization without image guidance, the indications to apply virtual isocenter for alignment, and the idea point for establishing virtual isocenter, which should improve the accuracy of frameless radiosurgery by phantom study. There should be more uncertainties when performing frameless radiosurgery in true human. Further studies should be designed to explore more details in this scenario for improving the reliability and accuracy of frameless radiosurgery.

## V. CONCLUSIONS

Set up, immobilization, and verification are important issues for an accuracy of frameless radiosurgery. However, thermoplastic masks are not sufficiently for position representation. Image guidance is necessary to improve the accuracy. Image guidance depends on image information. Under certain scenarios, when the treatment isocenter is less than 30 mm from the skull edge, the image information will not be satisfactory for alignment by image fusion. Alignment according to virtual isocenter should be used to improve the accuracy. The virtual isocenter is suggested to be set up as caudally as possible, and the sella of skull should be an ideal point.
